# Modulation effects of the East Asian winter monsoon on El Niño-related rainfall anomalies in southeastern China

**DOI:** 10.1038/s41598-018-32492-1

**Published:** 2018-09-20

**Authors:** Tianjiao Ma, Wen Chen, Juan Feng, Renguang Wu

**Affiliations:** 1 Center for Monsoon System Research, Institute of Atmospheric Physics, Chinese Academy of Sciences, Beijing, 100190 China; 2 College of Earth and Planetary Sciences, University of Chinese Academy of Sciences, Beijing, 100049 China

## Abstract

The present study investigates the modulation of the East Asian winter monsoon (EAWM) in the impacts of El Niño on the wintertime rainfall anomalies in southeastern China. Here, the variability of the EAWM that is independent of ENSO is considered, which is referred to as EAWM_res_ with a strong EAWM_res_ denoting anomalous northerly winds. Results demonstrate that strong (weak) EAWM_res_ weakens (enhances) the positive rainfall anomalies in southeastern China induced by El Niño, because anomalous downward motion over the western North Pacific (WNP) associated with El Niño is weakened (strengthened) by strong (weak) EAWM_res_. The modulated convective activity over the WNP, on the one hand, changes the anomalous local Hadley circulation associated with El Niño. When El Niño is combined with strong (weak) EAWM_res_, anomalous local Hadley circulation is weak (strong) and the resultant anomalous upward motion is weak (strong) over southeastern China, leading to small (larger) positive rainfall anomalies there. On the other hand, the modulated WNP convective activity induces different low-level atmospheric responses to El Niño. During an El Niño winter with strong (weak) EAWM_res_, the weak (strong) anomalous suppressed convection produces a weak (strong) and insignificant (significant) anomalous low-level WNP anticyclone, resulting in correspondingly insignificant (significant) rainfall anomalies in southeastern China. Results from a linear baroclinic model further suggest that the different low-level atmospheric responses over WNP are mainly induced by different diabatic heating associated with El Niño under different EAWM_res_ conditions.

## Introduction

The East Asian monsoon system dominates the seasonal variation of climate over East Asia and favors abundant rainfall in summer but infrequent precipitation in winter^1–5^. Many of these previous studies have been focused on the summer precipitation because it is of great importance to the agriculture and industry^6–10^. However, the wintertime precipitation in East Asia has received less concern. During the East Asian winter, the climatological rainfall belt lies in southeastern China and southern Japan, where has a concentration of population, advanced infrastructure, developed industry and large scope of agriculture. Strong interannual variability of the rainfall belt often caused large societal and economic impacts^11–13^. For example, the extreme severe snowstorm in the winter of 2007/2008 in southeastern China had damaged 8381 power towers and 2018 transformer stations, and deprived 129 lives^13–15^. Severe drought occurred persistently from the winter of 2011 to the succeeding spring in southern China, leading to 1.09 billion economic loss^16^. Therefore, it is getting ever more important to understand the interannual variability of the wintertime rainfall in southeastern China.

The winter precipitation in East Asia is affected by many factors. For example, the East Asian winter monsoon (EAWM) system^11,12,17^, El Niño-Southern Oscillation (ENSO)^18–20^, the Tibetan Plateau thermal state^16^, the warm pool ocean heat content^21^, and low-latitude systems including the Bay of Bengal trough and the Northwestern Pacific subtropical high^12,19^, etc. Among the factors that impact the southeastern China rainfall in winter, the EAWM and ENSO play the most important role on interannual time scales. The EAWM features with northeasterlies near the surface along the coastal eastern Asia^22–27^. A weak EAWM is often accompanied by southeasterly wind anomalies along the coast of East Asia, which transport warm and moist air from the tropical ocean northward to southeastern China, further induces moisture convergence and results in above-normal rainfall there, and vice versa^12,28,29^. ENSO is the most important tropical air-sea couple system on interannual time scales, which can influence the climate in the East Asia region via Gill-type response in terms of tropical Rossby waves^20,30,31^. During an El Niño (La Niña) winter, positive (negative) rainfall anomalies in southeastern China are often observed due to an anomalous lower-tropospheric anticyclone (cyclone) over the western North Pacific (WNP) induced by El Niño (La Niña)^18–20,30,32^.

The interaction between ENSO and the EAWM makes their impacts on the wintertime precipitation in southeastern China complex. On the one hand, an El Niño (La Niña) is often accompanied by a weak (strong) EAWM as anomalous southerly (northerly) winds to the northwest of an El Niño-induced anticyclone (cyclone) impair (favor) the southward penetrating of cold waves^19,20,30^. On the other hand, a strong EAWM can act as a trigger for an El Niño event through inducing westerly wind anomalies over the equatorial western Pacific^33^. The respective impacts of ENSO and EAWM on the wintertime rainfall in China has been investigated by Zhou and Wu^29^. They found that the independent EAWM dominantly exerts its impact on rainfall anomalies in eastern China, while the independent ENSO dominantly in southern China. ENSO-induced climate anomalies are not steady worldwide^34–36^. Especially, recent studies have noticed that the relationship between ENSO and the climate variability in East Asia is not stable. This unsteady relationship may be caused by many factors, such as different flavors of ENSO and extra-tropical influences. Compared with the traditional eastern Pacific El Niño, the central Pacific El Niño (also known as El Niño Modoki) tends to cause less precipitation anomalies in southern China from winter to the subsequent summer due to a weaker and southwestward shift of WNP anticyclone^37,38^. Wang *et al*.^39^ showed that ENSO has a significant (hardly any) impact on the EAWM during a negative (positive) phase of Pacific Decadal Oscillation (PDO). Jia and Ge^40^ reported that the influence of the EAWM as well as ENSO on the wintertime precipitation in southeastern China is strong during the period of 1980–1998, but weak in 1999–2015.

It is widely accepted that ENSO exerts its impacts on the wintertime rainfall in southeastern China through yielding an anomalous lower tropospheric anticyclone (cyclone) over the WNP near the Philippine Sea^20,30^. However, the WNP anticyclone (cyclone), as well as the relationship between ENSO and the climate in southern East Asia, can be modulated by extra-tropical factors. For instance, Kim *et al*.^41^ reported that a negative PDO or a positive Atlantic Multidecadal Oscillation (AMO) phase tends to drive a westward shift of the WNP anticyclone, resulting in a stronger connection between ENSO and the climate of East Asia. It is difficult to recognize the influence of EAWM on the WNP anticyclone (cyclone) associated with ENSO due to the interaction between ENSO and the EAWM. However, there is evidence that a weak (strong) EAWM_res_, which represents the variation of the EAWM that is linear independent of ENSO, is associated with an anomalous anticyclonic (cyclonic) circulation over the WNP near the Philippine Sea^36^. Feng and Chen^42^ demonstrated that EAWM_res_ modulates the impacts of El Niño (La Niña) on the following East Asian summer monsoon through changing the intensity and domain of the El Niño-induced western North Pacific anticyclone (WNPAC). Hence, we wonder whether the EAWM could interfere with the impacts of ENSO on wintertime rainfall in southeastern China. This study aims to address this issue. Additionally, it is reported that the relationship between La Niña and the winter rainfall anomalies in China is insignificant^43^. Hence, we will focus on the rainfall anomalies induced by El Niño in this study.

The rest of the paper is organized as follows: Section 2(a) documents roles of EAWM_res_ in modulating the impacts of El Niño on the wintertime rainfall anomalies in China. In section 2(b) and (c), possible mechanisms are elucidated by the observational analysis and the experiments with a linear baroclinic model (LBM), respectively. The summary is provided in section 3. Descriptions of data, methods, and the linear baroclinic model are given in section 4.

## Results

### Rainfall anomalies associated with El Niño and EAWM_res_

**Figure 1:**
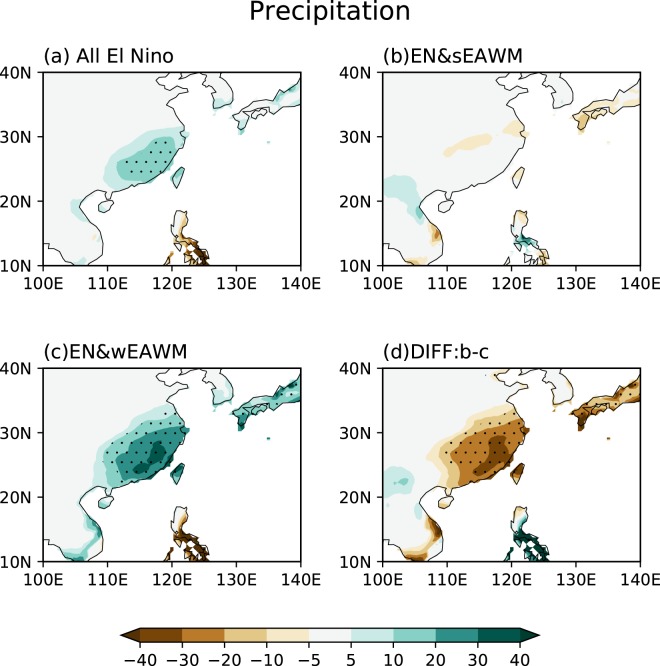
Composite wintertime mean (DJF) rainfall anomalies during (**a**) all El Niño events, (**b**) El Niño & Strong EAWM_res_ events (EN&sEAWM), (**c**) El Niño & weak EAWM_res_ (EN&wEAWM) events and (**d**) the difference between (**b** and **c**). The unit of rainfall anomalies is mm/month. The dotting indicates significance at the 95% level. This Figure is created by the Matplotlib^54^ v2.0.2 (10.5281/zenodo.573577).

To investigate the modulation effects of EAWM_res_ on the rainfall anomalies in China associated with El Niño, Fig. 1 presents the composite rainfall anomalies in all the El Niño winters, the El Niño winters with strong EAWM_res_ (hereafter, EN&sEAWM), and the El Niño winters with weak EAWM_res_ (hereafter, EN&wEAWM). The pattern of rainfall anomalies in all the El Niño events (Fig. 1a) is characterized by positive values in southeastern China, which is consistent with the well-known results that El Niño tends to increase the East Asian winter rainfall^12,18,19,28–30,32,44^. However, in the group of EN&sEAWM, no positive rainfall anomaly is observed in southeastern China, but only a weak negative rainfall belt is seen along the lower reaches of the Yangtze River (Fig. 1b). In contrast, in the group of EN&wEAWM (Fig. 1c), anomalous rainfall distribution bears a close resemblance to that in the group of all the El Niño winters except that the positive rainfall anomalies are stronger and extend northwestward. The difference of rainfall anomalies between the EN&sEAWM and EN&wEAWM group (Fig. 1d) indicates that the EAWM_res_ plays an important role in the impact of El Niño on the rainfall anomalies in southeastern China. Strong (weak) EAWM_res_ tends to suppress (enhance) anomalous rainfall response to El Niño in southeastern China.

Another East Asian monsoon index defined by Zhang *et al*.^20^ is employed to confirm the robustness of our results. This East Asian monsoon index is calculated by the 3-month running mean of meridional wind anomalies at 850 hPa averaged over the area of 20–30°N, 110–130°E and shows a good relationship with El Niño. By using this index, very similar results are obtained compared to Fig. 1 (figure not shown). In addition, to make sure that the differences of rainfall anomalies between the two El Niño groups are caused by the EAWM variability rather than asymmetric response between the super and weak El Niño events, we further removed several weak (1970, 1977, 1978, and 2007) and super El Niño (1973, 1983, and 1998) cases from the corresponding groups. Again, very similar results are obtained with those shown in Fig. 1 (figure not shown). Hence, the EAWM_res_ has a significant modulation effect on the winter rainfall anomalies in China associated with El Niño.

### Possible Mechanism

#### Results from observations

**Figure 2:**
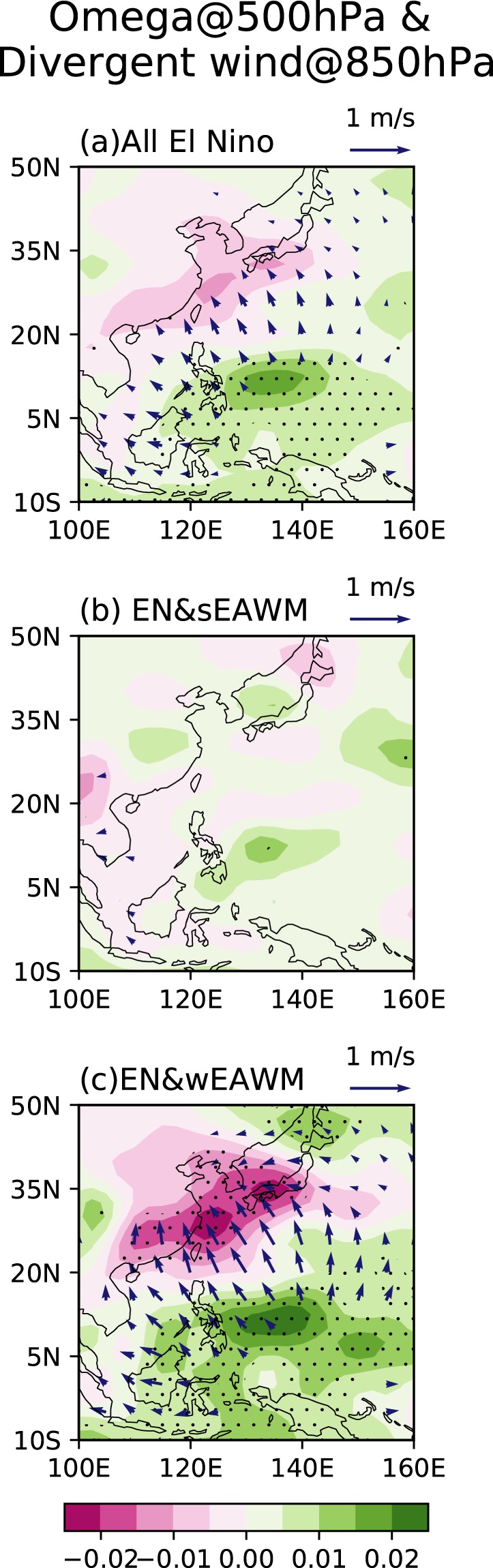
Composite winter mean (DJF) 500 hPa vertical p-velocity anomalies (color; unit:  Pa/s) and 850 hPa divergent winds (vector; unit: m/s) in groups of (**a**) all El Niño (**b**) El Niño & strong EAWM_res_ (EN&sEAWM) and (**c**) El Niño & weak EAWM_res_ (EN&wEAWM). The dotting indicates that the anomalous vertical motion is significance at the 95% level. Vectors only exceed the 95% significance level are shown. This Figure is created by the Matplotlib^54^ v2.0.2 (10.5281/zenodo.573577).

During an El Niño event, suppressed convective activities and associated diabatic cooling over the tropical western Pacific play important roles in linking El Niño to the rainfall anomalies in East Asia^19,20,30,32^. Zhang *et al*.^20^ first proposed the explanation of the WNPAC as a Rossby wave response to the suppressed convection over the tropical western Pacific. Recently, Zhang *et al*.^45^ further reviewed the association of El Niño with the WNPAC and the possible mechanisms. On the one hand, the anomalous subsidence over the tropical western Pacific results in a weakened local Hadley cell which can affect the meridional East Asian monsoon circulation. On the other hand, a WNPAC is generated in response to the diabatic cooling over the tropical western Pacific, which may transport moisture to southern China and increase rainfall there. To understand how the EAWM_res_ modulates the impacts of El Niño events, we diagnosed the 500 hPa vertical p-velocity motion and 850 hPa divergent winds as shown in Fig. 2. In all the El Niño winters, an anomalous sinking over east of the Philippines corresponds to the decreased convective activities there, which is consistent with previous studies^32^. Meanwhile, anomalous rising and correspondent convergent winds in the lower troposphere are seen over southeastern China (Fig. 2a). This result implies that the local Hadley circulation is disturbed by the El Niño events, with anomalous ascending flow over southeastern China and descending flow over the western Pacific. Hence, the upward motion and low-level convergence contribute to the positive rainfall anomalies in southeastern China as shown in Fig. 1a. However, this anomalous local Hadley circulation is substantially changed after considering the EAWM_res_. In the group of EN&sEAWM, the El Niño-induced low-level anomalous divergent winds around the Philippines and the associated downward motions are greatly weakened (Fig. 2b). There are nearly no upward motions as well as convergence observed over southeastern China and no positive rainfall anomalies induced in the EN&sEAWM cases. In contrast, in the group of EN&wEAWM, the local Hadley circulation tends to become much stronger compared to that in the group of EN&sEAWM. Hence, more significant positive rainfall anomalies in southeastern China are induced in the EN&wEAWM winters (Fig. 2c). Comparing Fig. 2b with Fig. 2c, it is obvious that the EAWM_res_ can modulate the El Niño-related convective activities around the Philippines. Note that strong (weak) EAWM_res_ often carries northerly (southerly) wind anomalies to penetrate southward to lower latitudes^36,42^. Therefore, convective activity around the Philippines is enhanced (suppressed) by strong (weak) EAWM_res_, which weakens (intensifies) the El Niño-induced suppressed convection around the Philippines.

**Figure 3:**
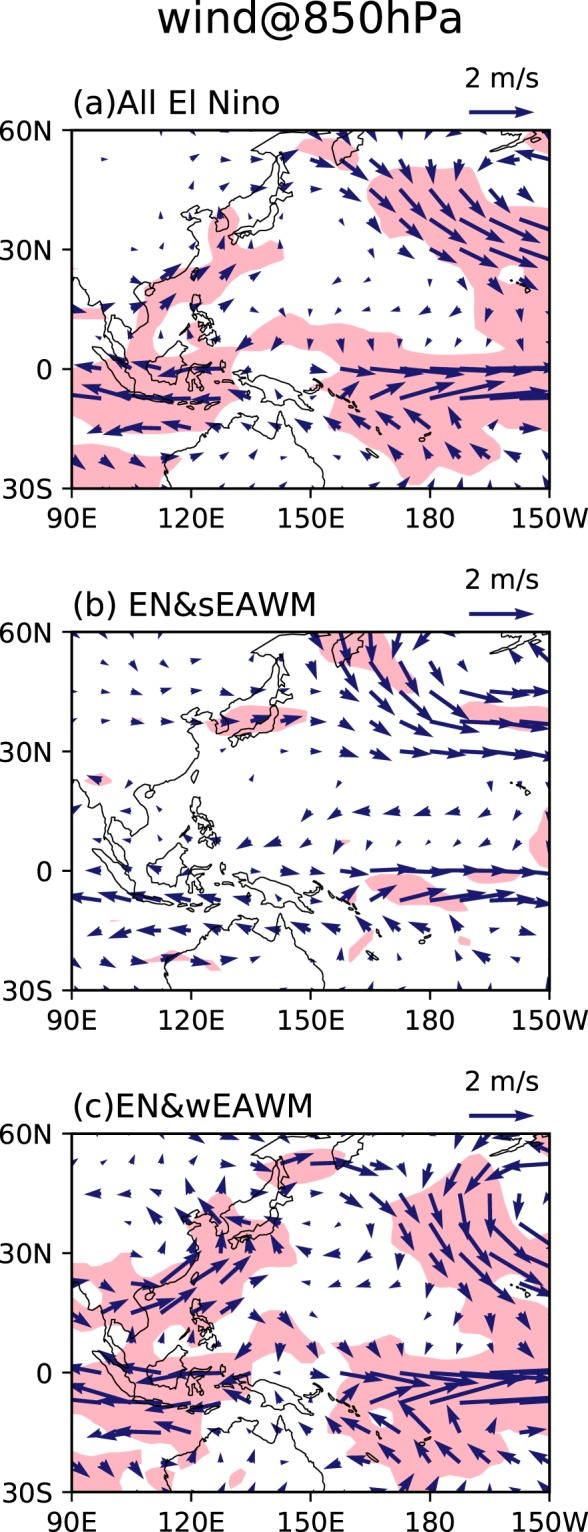
As in Fig. 2, but for 850 hPa wind (vector, m/s). Shading indicates that either the meridional wind or the zonal wind exceeds the 95% significance level. This Figure is created by the Matplotlib^54^ v2.0.2 (10.5281/zenodo.573577).

The WNPAC is further presented in Fig. 3 to check the different responses of horizontal atmospheric circulation over East Asia to the diabatic cooling over the tropical western Pacific. In the group of all the El Niño winters, an obviously anomalous WNPAC is observed, which is again consistent with previous studies^20,30–32^. However, when an El Niño event happens with a strong EAWM_res_, the WNPAC is much weakened (Fig. 3b). This is because the strong northerly wind anomalies along the East Asian coast associated with the strong EAWM_res_ weaken the southerly wind anomalies to the west flank of the anomalous WNPAC. In contrast, a much stronger WNPAC appears when an El Niño event happens with a weak EAWM_res_ (Fig. 3c). Especially, much stronger southerly wind anomalies to the west flank of the anomalous WNPAC may transport plentiful moisture to southeastern China, and increase the positive rainfall anomalies in southeastern China as shown in Fig. 1c.

### Possible Mechanism

#### Results from a LBM

**Figure 4:**
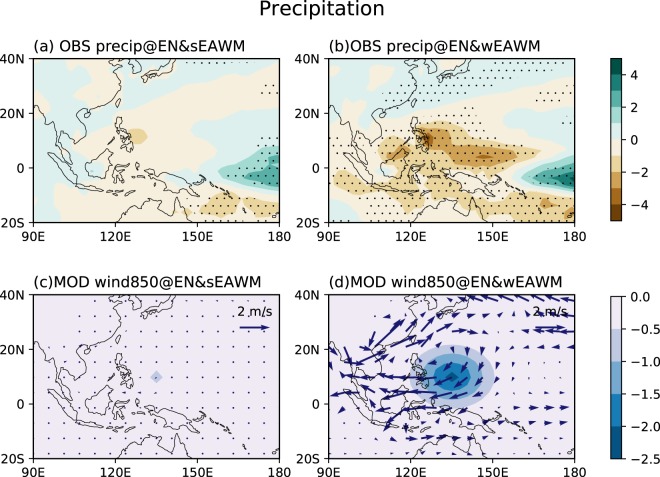
Composite winter mean (DJF) rainfall anomalies during (**a**) El Niño & strong EAWM_res_ events (EN&sEAWM) and (**b**) El Niño & weak EAWM_res_ (EN&wEAWM) events based on PREC reanalysis dataset (Unit: mm/day). Response of 850 hPa winds (vector; m/s) to the heat source over the WNP in the case of (**c**) EN&sEAWM and (**d**) EN&wEAWM. The 11 day average from day 15–25 is shown. Color filling in (**c** and **d**) represent the horizontal distribution of the specific heating (K/day) at σ = 0.45 level. The dotting in (**a** and **b**) indicates that the rainfall anomaly is significant at the 95% level. This Figure is created by the Matplotlib^54^ v2.0.2 (10.5281/zenodo.573577).

We further illustrate that the difference in WNPAC between the EN&sEAWM and EN&wEAWM winters is induced by different convective activities over the WNP through a simple linear baroclinic model^46^. Two experiments are designed through prescribing different diabatic forcing associated with the EN&sEAWM and EN&wEAWM cases, respectively. The basic state is prescribed as the winter mean for the period of 1948–2015 using the NCEP/NCAR reanalysis data. The external forcing is set as the diabatic heating estimated from the observational precipitation anomalies (Fig. 4a,b). The vertical profile of the heating has a Gamma distribution which peaks at σ = 0.45 (about 400 hPa). The minimum negative heating over the WNP at σ = 0.45 for the EN&sEAWM (EN&wEAWM) winters is −0.64 (−2.26) K/day, which is approximately equivalent to the latent heating released by precipitation with the value of −1 (−3.5) mm/day. In the horizontal, the heating has an idealized elliptical profile, which mimics the position and strength of observational rainfall anomalies (Fig. 4c,d). Hence, the intensity and spatial distribution of diabatic forcing in the model are basically in accord with the latent heating released by the observational precipitation. The length of time integration is set as 30 days, and the results shown here is the mean from day 15 to 25. This is because the atmospheric response is equilibrated roughly after day 10, but the baroclinic waves would rapidly grow and the model intergration would blow up after around day 30.

Figure 4c,d present the atmospheric responses of 850 hPa winds to the prescribed diabatic heating in the EN&sEAWM and EN&wEAWM experiments, respectively. In the EN&sEAWM experiment, there is no low-level anticyclonic response over the WNP. This agrees with the weakened diabatic forcing around the WNP as specified. In contrast, in the EN&wEAWM experiment, an anticyclone over the WNP is clearly seen, which is induced by the strong diabatic forcing as specified. The low-level atmospheric responses over the WNP in the two experiments are generally in accordance with the observational results. The westerly winds along the equatorial western Pacific in both of the EN&sEAWM and EN&wEAWM experiments are weaker than those in the observation, which might be related to the absence of eastern Pacific heating in the model. Therefore, the differences in the WNPAC between the EN&sEAWM and EN&wEAWM winters are further confirmed to arise from the different diabatic heating over the WNP.

## Summary and Discussion

This study investigated the modulation effect of the EAWM on the relationship between El Niño and the rainfall anomalies in southeastern China through composite analysis and a simple linear baroclinic model simulation. The El Niño winters were divided into two groups based on the intensity of EAWM_res_: the El Niño with strong EAWM_res_ (EN&sEAWM) and the El Niño with weak EAWM_res_ (EN&wEAWM).

An El Niño event often causes above-normal winter rainfall in southeastern China. However, the rainfall anomalies associated El Niño show totally different features after considering the condition of EAWM_res_. Nearly no significant rainfall anomalies are observed in southeastern China in the EN&sEAWM winters. In contrast, in the EN&wEAWM winters, positive precipitation anomalies are increased and cover a larger domain of southeastern China. The magnitude of precipitation anomalies in the EN&wEAWM winters is about twice of that in the all El Niño winters. The result indicates that the strong (weak) EAWM_res_ weakens (enhances) the impacts of El Niño on the winter rainfall in southeastern China.

The effect of EAWM_res_ on the rainfall is suggested to be induced by its modulation on the anomalous downward motion around the WNP associated with El Niño. Strong EAWM_res_ carries northerly wind anomalies to penetrate southward deeply and causes anomalous upward motion over the WNP, whereas weak EAWM_res_ has the opposite effect on the WNP convective activity. Hence, the El Niño-induced downward motion over the WNP is weakened (strengthened) by strong (weak) EAWM_res_ in the case of EN&sEAWM (EN&wEAWM). This modulated convective activity over the WNP tends to influence the local Hadley circulation associated with El Niño. In the EN&sEAWM winters, the local Hadley circulation becomes much weaker. In contrast, in the EN&wEAWM winters, the local Hadley circulation becomes much stronger. The changes in the local Hadley circulation induce much stronger anomalous upward motion in the EN&wEAWM winters than the EN&sEAWM winters. Hence, significantly excessive rainfall anomalies are observed in the EN&wEAWM winters.

On the other hand, the convective activities modulated by the EAWM_res_ during the El Niño events yield different responses of the WNPAC. In the EN&sEAWM winters, the WNPAC response is weak due to weak convective anomalies over the WNP, leading to weak rainfall response. In the EN&wEAWM winters, strengthened suppressed convection over the WNP induces a much stronger WNPAC, which corresponds to excessive rainfall in southeastern China by transporting plentiful moisture.

The effect of anomalous convective heating over the WNP is further validated by experiments with a dry linear baroclinic model. The result demonstrates that the intensity of low-level anomalous WNPAC is dominantly controlled by the magnitude of anomalous diabatic heating over the WNP. The numerical results are largely consistent with the observations, suggesting that the difference in the diabatic heating anomalies in the EN&sEAWM and EN&wEAWM winters account for the different atmosphere circulation anomalies over the WNP.

## Data, Methods and Model Introduction

The Climatic Research Unit (CRU) Time-series (TS) version 4.00 rainfall data is employed in this study, which has a horizontal resolution of 0.5° latitude by 0.5° longitude and spans the time period from 1901 to 2015^47^. The CRU data are provided by the Centre for Environmental Data Analysis from their web site http://catalogue.ceda.ac.uk. The monthly mean atmospheric data used in this study are from the National Centers for Environmental Prediction (NCEP) /National Center for Atmospheric Research (NCAR) and available at the website of NOAA/OAR/ESRL PSD https://www.esrl.noaa.gov/psd. This NCEP/NCAR dataset has a 2.5° × 2.5° horizontal resolution and 17 vertical levels extending from 1000 to 10 hPa and covers the period from 1948 to the present^48^. The monthly mean Sea Surface Temperature (SST) data on 1° × 1° grids are provided by the Met Office Hadley Center (https://www.metoffice.gov.uk), covering the period from the year of 1870 to present^49^. The National Oceanic and Atmospheric Administration (NOAA) reconstructed precipitation (PREC) data, provided also by the NOAA/OAR/ESRL PSD, are used to analyze the rainfall anomalies over oceans, which is constructed on a 2.5° × 2.5° latitude/longitude grid across the global spanning from 1948 to the present^50^. The time period of 1948–2015 that all the datasets cover is considered in this study. To exclude the impacts of interdecadal change, the variability longer than 7 years has been removed from the original datasets through a Lanczos filter^51^.

The historical El Niño/La Niña episodes selected in this study are based on the criterion defined by the Climate Prediction Center (CPC). An El Niño (La Niña) event is identified when the Oceanic Niño Index (ONI) is greater (less) than 0.5 °C (−0.5 °C) for 5 consecutive overlapping seasons, and the ONI is defined as 3-month-mean of the SST anomalies over the Niño 3.4 region (5N°–5°S, 170°W–120°W). Therefore, 24 El Niño events during the period of 1948–2015 are finally picked out.

To isolate the EAWM component that is independent of ENSO influence, we followed the method proposed by Ma *et al*.^52^. Specifically, an EAWM index, which is defined by the 850 hPa meridional wind anomaly averaged over the area (20–40°N, 100–140°E)^27^, is used to estimate the strength of EAWM. As the El Niño episode is selected based on the SST anomalies in Nino 3.4 region, the ENSO signal is determined by the Nino 3.4 index here. Then, the ENSO-related EAWM (EAWM_EN_) index is computed by a linear regression of EAWM index upon the Nino3.4 index. The ENSO-unrelated EAWM index, which is referred to as EAWM_res_, is finally obtained by the total EAWM index minus the EAWM_EN_ index. Here, a positive (negative) EAWM_res_ index represents a weak (strong) EAWM_res_ winter. On the basis of the EAWM_res_ index, the El Niño events are sorted into two groups (Table 1): (1) El Niño with strong EAWM_res_ (EN&sEAWM); and (2) El Niño with weak EAWM_res_ (EN&wEAWM).

**Table 1:** Distribution of the El Niño events based on the strength of EAWM_res_.

Groups	Years
El Niño - Strong EAWM_res_	1952 1953 1966 1970 1977 1978 1987 1988 1992 1995 2007 2015
El Niño - Weak EAWM_res_	1954 1958 1959 1964 1969 1973 1980 1983 1998 2003 2005 2010

Here, 1952 indicats the boreal winter mean of December 1951 to Feburay 1952.

The Linear Baroclinic Model (LBM) Package is used in this study, which is developed by the Center for Climate System Research (CCSR), University of Tokyo and the National Institute for Environmental Studies (NIES)^46,53^. The experiments are set in a T42 resolution on horizontal and 20 levels in the vertical on σ surface. To analyze the steady atmospheric response to a diabatic heating anomaly, a dry model and time integration method is employed. The basic state and the external forcing in the LBM are computed from the NCEP/NCAR reanalysis data and PREC precipitation data, respectively.
